# Headaches in Multiple Sclerosis Patients Might Imply an Inflammatorial Process

**DOI:** 10.1371/journal.pone.0069570

**Published:** 2013-08-05

**Authors:** Jan Möhrke, Peter Kropp, Uwe K. Zettl

**Affiliations:** 1 Institute of Medical Psychology and Medical Sociology, Medical Faculty, University of Rostock, Rostock, Germany; 2 Department of Neurology, Medical Faculty, University of Rostock, Rostock, Germany; University of Muenster, Germany

## Abstract

Recent studies on Multiple Sclerosis (MS) pathology mention the involvement of “tertiary B cell follicles” in MS pathogenesis. This inflammatory process, which occurs with interindividually great variance, might be a link between MS pathology and headaches. The aim of this study was to detect the prevalence of headaches and of subtypes of headaches (migraine, cluster, tension-type headache [TTH]) in an unselected MS collective and to compile possibly influencing factors. Unselected MS patients (n = 180) with and without headache were examined by a semi-structured interview using a questionnaire about headache, depression and the health status. Additionally clinical MS data (expanded disability state score [EDSS], MS course, medication, disease duration) were gathered. N = 98 MS patients (55.4%) reported headaches in the previous 4 weeks. We subsequently grouped headache patients according to the IHS criteria and detected 16 (16.3%) MS patients suffering from migraine (migraine with aura: 2 [2%]; migraine without aura: 14 [14.3%]), 23 (23.5%) suffering from TTH and none with a cluster headache. Thus, headaches of 59 (60.2%) MS patients remained unclassified. When comparing MS patients with and without headaches significant differences in age, gender, MS course, physical functioning, pain and social functioning occurred. MS patients with headaches were significantly younger of age (p = 0.001), female (p = 0.001) and reported more often of a clinically isolated syndrome (CIS) and relapsing/remitting MS (RRMS) instead of secondary chronic progressive MS (SCP). EDSS was significantly lower in MS patients suffering from headaches compared to the MS patients without headaches (p = 0.001). In conclusion headache in MS patients is a relevant symptom, especially in early stages of the MS disease. Especially unclassified headache seems to represent an important symptom in MS course and requires increased attention.

## Introduction

Multiple sclerosis (MS) is a chronic inflammatory process, which aetiology is not yet completely understood [1]. Currently a complex interplay of genetic and environmental associated factors is considered [2], [3]. Recent studies on the pathology of MS showed that the meninges are involved in the pathogenesis of MS with “tertiary B-cell follicles” manifestation in the gyri [4], [5], [6]. The B-cell-follicles discovered by Magliozzi et al. [4], [5], [6] transfer their antibodies to manifest MS lesions leading to an inflammatory process, which is known to be interindividually different pronounced. As meningeal irritations are known to cause headaches [7], this meningeal inflammatory process could be a pathomorphological substrate of headaches in MS.

The International Headache Society [8] distinguishes between primary, secondary, and other headaches. We mainly focussed on the primary headaches like migraine, tension type headache (TTH) and cluster headaches. Migraine is a pulsating, often one-sided headache with a duration of 4 to 72 hours [8] with a prevalence of 10% in the normal population [9]. The aetiology and pathogenesis are still subject to research. Experimental and clinical data imply cortical and subcortical factors in pathophysiology of the migraine attack [10], [11]. Especially the trigemino-vascular system (TVS) plays a central role in initiating headaches. The TVS controls vessels of dura and pia regions and serves simultaneously as a mediator to cortical regions. The prevalence of migraine in MS patients varies widely between 43,3% [12] up to 71,8% [13].

TTH is characterized as a continual nagging-pressing pain with a life time prevalence of 30–70% [8]. Similarly to migraine, aetiology and pathogenesis are not yet completely understood [14], but central mechanisms are obvious. Similarly to migraine, TTH in MS patients varies between 12,2% [15] up to 55,2% [12].

Cluster headaches are described as a sharp paroxysmal unilateral pain of severe intensity associated with eye pain [16]. Its prevalence varies between 0.4%–0.6% in the normal population [17]. Currently a central dysregulation of the hypothalamus is discussed as an influencing factor [17], [18]. Studies on cluster headaches in MS patients are rare, thus a prevalence of cluster headache in MS patients cannot be exactly specified. An exact finding might probably be difficult due to the low prevalence rate in the normal population.

An overview of recent studies on headaches in MS patients is shown in table 1.

**Table 1:** Compilation of headache subtypes in MS.

Study	n	Mig	TTH	M+TTH	TN	CH	Cluster	SH	NC
[12]	67	29 (43.3)	37 (55.2)				1 (1.5)		
[26]	154	95 (61.4)	39 (25.3)	20 (13)			0	0	0
[41]	122	100 (82.0)	17 (13.9)						5 (4.1)
[15]	74	46 (62.2)	9 (12.2)			8 (10.8)		11 (14.9)	
[27]	101	20 (19.8)	28 (27.7)				0	4 (4)	6 (5.9)
[29]	6	5 (83.3)	1 (16.7)						
[28]	295	121 (41)	143 (48.5)	0	31 (10.5)		0	0	0
[42]	131	94 (71.8)	37 (28.2)				0	0	0

N  =  patients with MS and headaches in the study, bracket terms: percentage.

Mig  =  migraine, TTH  =  tension type headache, Mig+TTH  =  migraine and tension type headache, TN  =  trigeminal neuralgia, CH  =  chronic headache, SH  =  secondary headache, NC  =  not classified by his criteria.

The observation of migraine-like headaches in subtypes of MS implies a common pathway of pain modulation both in migraine and in MS patients. We hypothesize that headaches may be associated with MS, especially with the RRMS. Up to now technical and methodological possibilities to elucidate this association are missing.

The aim of our study was to calculate the general prevalence of primary headaches and of the different subtypes (migraine, cluster, TTH) in an unselected collective of MS-patients. As a second aim we wanted to isolate factors influencing headaches in MS.

## Methods

All MS-patients were examined in a semi-structured interview with three questionnaires to classify headaches according to the IHS-criteria and to record the emotional state by Beck Depression Inventory (BDI, [19]) and quality of life (“Short Form”, SF-36, [20]).

### Ethics statement

The study was approved by the ethics committee of the Medical Faculty of the University of Rostock, Germany.

### Subjects

Unselected MS-patients (n = 180) with and without headache were examined. All patients were interviewed in the Department of Neurology at the University of Rostock, Germany. The selection was carried out continuously, neither gender nor age-related. In all examined patients the diagnosis was clinically confirmed using the McDonald criteria [21]. None of the patients showed secondary diseases besides their MS that would suggest a secondary headache like acute intracranial haemorrhage, pathogen-induced inflammation, a brain tumour, degenerative changes in the bony skull, or hydrocephalus.

### Questionnaires

The questionnaires were completed by clinical data of the MS (course, EDSS, onset and duration of disease, medication). MS was classified using the McDonald criteria from 2005 [21]. To classify headaches we used a structured headache questionnaire which checked the criteria according to the International Headache Society [8] for primary headaches. These were classified into migraine with and without aura, tension-type headache, or cluster-headache. For migraine without aura, the questionnaire reveals a sensitivity of 0,86 and a specificity of 0,51. Regarding migraine with aura, the questionnaire has a sensitivity of 0,71 and a specificity of 0,95. For the tension type headaches the questionnaire reveals a sensitivity of 0,57 and a specificity of 0,93. In this study we did not distinguish between migraine with and without aura.

Emotional state was recorded by the Beck's Depression Inventory [22]. The BDI allows to reveal a possible or manifest depression. General health status was recorded by the “Short-Form 36-questionnaire” (SF-36, [20]). SF-36 includes eight dimensions, which are summarized by a “physical health” and a “mental health” score. The eight SF-36 concepts involve “physical functioning”, “role limitations due to physical health”, “bodily pain”, “general health perceptions”, “vitality”, “social functioning”, “role limitations due to emotional problems” and “general mental health”.

### Classification

Based on the clinical data and the questionnaires, we compiled MS-course, -medication, and –duration with headaches diagnosis, emotional state, and quality of life.

### Statistics

Group comparisons were performed using Student's t-tests [24]. Categorical variables were compared using the Chi-square test of Pearson [23]. For the variables “medication of MS” and “headache medication” we used univariate analyses of variance (ANOVA; [24]). Influencing factors were performed using the binary logistic regression [24]. Unless otherwise specified, significance level was set to 5%. All calculations were performed by Statistical Package for Social Sciences –software (SPSS), Version 18.

## Results

### 1. Patients characteristics

We examined 125 women (69.4%) and 55 men (30.6%). Mean age was 43.9 years (SD 13.1 years). Patients suffered from MS for 12.3 years (SD 8.8 years). The most common course of MS was the relapsing-remitting MS (RRMS, 86, 47.8%), the second most common form was the secondary chronic progressive MS (67, 37.2%). A primary chronic progressive MS (PPMS) was observed in 24 patients (13.3%) and a clinically isolated syndrome (CIS) was diagnosed in 3 patients (1.7%).

### 2. Medication

As disease-modifying drug (DMD)-medication corticosteroids were applied to 69 MS patients (48.3%), followed by beta-interferon preparations in 32 MS patients (17.8%). 24 MS patients (13.3%) were treated with mitoxantrone, 21 MS patients (11.7%) received intravenous immunoglobulin (IVIG). 14 MS patients (7.8%) were treated with monoclonal antibodies (natalizumab, rituximab), and 8 MS-patients (4.4%) received glatiramer acetate. During our study 12 MS patients (6.7%) received no immunmodulatory therapy.

### 3. Severeness of multiple sclerosis

The mean degree of MS severeness, operationalized by the EDSS, was 3.6 (SD 2.3), the mean BDI-score of all MS patients was 6.2 (SD 4.3). Sociodemographic and neurological parameters of our study are shown in detail in table 2.

**Table 2:** Socio-demographic and neurological parameters of MS patients.

	N	percentage	mean	SD
Patients	180			
Age *(in years)*			43.9	13.1
- female	125	69.4		
- male	55	30.6		
Disease duration of MS *(years)*			12.3	8.8
- RRMS	86	47.8		
- SCP	67	37.2		
- PPMS	24	13.3		
- CIS	3	1.7		
Beta-Interferons	32	17.8		
Glatirameracetat	8	4.4		
IVIG	21	11.7		
Mitoxantron	24	13.3		
Monoclonal Antibodies	14	7.8		
- cyclic Steroids	69	38.3		
- no medication	12	6.7		
BDI			6.2	4.3
EDSS			3.6	2.3

SD = Standard deviation; MS = Multiple sclerosis; EDSS = Expanded Disability State Score; BDI = Beck's Depression inventary; RRMS = Relapsing/Remitting MS; SCP = Secondary chronic progressive MS; PPMS = Primary chronic progressive MS; CIS = Clinical isolated syndrome, IVIG  =  intravenous immunoglobulin.

### 4. Headache prevalence

98 (55.4%) MS patients reported headache during the previous 4 weeks, 82 (44.6%) denied any headache experience during this period. Using the IHS criteria we diagnosed 16 patients (16.3%) suffering from migraine (migraine with aura: 2 [2%]; migraine without aura: 14 [14.3%]), and 23 suffering from TTH (23.5%). No patient experienced a cluster headache according to the IHS-criteria. The remaining 59 patients (60.2%) were categorized as suffering from an unclassified headache, not fulfilling the IHS-criteria for migraine, TTH, or Cluster headache.

Afterwards, we compared the MS patients with headache to MS patients without headache and identified significant differences in sociodemographic and clinical data (table 3). The headache group differed significantly in age, gender, and the progressive form of MS, in medication, the EDSS, and in physical and social functioning. Patients with headache experience were significantly younger (p<0.001). Female patients (p<0.001) and patients less impaired in their motor function (EDSS, p<0.001) reported more headache. The secondary chronic progressive form of MS (SCP) was more frequent among non-headache patients while relapsing-remitting MS (RRMS) occurred more often among MS patients with headache.

**Table 3:** Comparison of sociodemographic and neurological parameters in MS-patients with and without headaches.

	headache		without headache		p
n	98		82		
Age: Years (SD)	41	(12.9)	47.4	(12.6)	0.001**
female	78	(79.6)	47	(57.3)	0.001**
male	20	(20.4)	35	(42.7)	
Disease Duration: years (SD)	11.2	(8.6)	13.7	(8.9)	0.056
- RRMS	56	(57.1)	30	(36.6)	0.042*
- SCP	29	(29.6)	38	(46.3)	
- PPMS	11	(11.2)	13	(15.9)	
- CIS	2	(2.0)	1	(1.2)	
Beta-Interferons	18	(18.4)	14	(17.1)	0.004**
Glatirameracetat	7	(7.1)	1	(1.2)	
IVIG	18	(18.4)	3	(3.7)	
Mitoxantron	13	(13.3)	11	(13.4)	
Monoclonal Antibodies	9	(9.2)	5	(6.1)	
cyclic Steroids	27	(27.6)	42	(51.2)	
no medication	6	(6.1)	6	(7.3)	
NSAR	56	(57.1)			
triptans	5	(5.1)			
combinations	10	(10.2)			
no medication	27	(27.6)			
BDI (SD)	6.7	(5)	5.5	(3.4)	0.078
EDSS (SD)	3	(2.2)	4.2	(2.3)	0.001***

SD = Standard deviation; MS = Multiple sclerosis; EDSS = Expanded Disability State Score; BDI = Beck's Depression inventory; RRMS = Relapsing/Remitting MS; SCP = Secondary chronic progressive MS; PPMS = Primary chronic progressive MS; CIS = Clinical isolated syndrome. IVIG  =  intravenous immunoglobulin. NSAR, triptans, combinations, no medication in case of headache treatment. * p<0.05; **: p<0.01, ***: p<0.001.

### 5. Headache and MS-medication

The DMD-medication was significantly different between the group suffering from headache or no headache (p = 0.004). Non-headache sufferers (NHS) were in half of the cases (51.2%) treated with glucocorticosteroids. On the contrary, only 27.6% in headache patients (HS) were treated with glucocorticosteroids (table 4). Compared to MS patients without headache those with headache were treated five times more often with glatiramer acetate (HS: 7.1% vs. NHS: 1.2%) and immunglobulines (HS: 18.4% vs. NHS: 3.7%).

**Table 4:** Comparison of sociodemographic and neurological parameters in MS-patients with and without analgesic medication.

	analgesic medication		without analgesic medication		p
Number	71		82		
Age: Years (SD)	39.7	(11.6)	44.4	(15.5)	0.008**
- Female	58	(81.7)	20	(74.1)	0.403
- Male	13	(18.3)	7	(25.9)	
Disease Duration: Years (SD)	10.5	(8.1)	13.2	(9.8)	0.191
RRMS	44	(62.0)	12	(44.4)	0.459
SCP	20	(28.2)	9	(33.3)	
PPMS	6	(8.5)	5	(18.5)	
CIS	1	(1.4)	1	(3.7)	
Beta-Interferons	14	(19.7)	4	(14.8)	0.5
Glatirameracetat	5	(7.0)	2	(7.4)	
IVIG	15	(21.1)	3	(11.1)	
Mitoxantron	9	(12.7)	4	(14.8)	
Monoclonal Antibodies	8	(11.3)	1	(3.7)	
cyclic Steroids	17	(23.9)	10	(37.0)	
no medication	3	(4.2)	3	(11.1)	
BDI (SD)	6.1	(4.4)	8.0	(6.1)	0.933
EDSS (SD)	2.8	(2.0)	3.5	(2.6)	0.028*

SD = Standard deviation; MS = Multiple sclerosis; EDSS = Expanded Disability State Score; BDI = Beck's Depression inventory; RRMS = Relapsing/Remitting MS; SCP = Secondary chronic progressive MS; PPMS = Primary chronic progressive MS; CIS = Clinical isolated syndrome, IVIG  =  intravenous immunoglobulin, *: p<0.05; **: p<0.01.

### 6. Headache and Quality of Life

Quality of life revealed significant differences in physical (p = 0.031) and social (p = 0.03) functioning and pain (p<0.001). Patients without headache were significantly more limited in their physical functioning than headache sufferers. However, they suffered less from pain and scored better in social functioning. Table 2 summarizes the sociodemographic and neurological results recorded for MS patients with and without headache.

Another important point of our study was to determine influencing factors for occurrence of headache and then the occurrence of specific types of headaches. The analysis of factors affecting the overall incidence of headache showed that younger age (p = 0.001), female gender (p = 0.001) and pain – operationalized by the SF-36– increased the possibility to suffer from headaches.

### 7. Migraine and MS-disability

The occurrence of migraine in MS patients was correlated reciprocally with EDSS-score: the lower the EDSS-score, the higher the possibility to suffer from migraine. Binary logistic regression reveals the following equation for the probability of the migraine occurrence in MS patients:

p = 1/(1+ e^−z^) with z = −1,343 −0,349× EDSS.

### 8. Tension-type headache

Suffering from TTH was correlated with the amount of bodily pain in SF-36. The higher the value in the scale “bodily pain”, the more probable was the occurrence of TTH in MS patients. Binary logistic regression reveals:

p = 1/(1+ e^−z^) with z = −0,69 −0,018× “bodily pain”.

## Discussion

The prevalence of headache varies widely in the general population. A large meta-analysis, which includes 107 studies of headaches worldwide, revealed a prevalence of headache of 47%, with a range from 1% to 91% [25]. In addition to the current prevalence of headaches, the lifetime prevalence was examined, i.e. the percentage of people who develop headaches anytime during their lifetime. Of course, this lifetime prevalence is higher than the current prevalence and is specified with 64% [25]. Women are affected to 73%, men to 68%. The calculated prevalence 64% does not reflect the expected average of 70.5%. This is due to the fact that some of the studies included only a prevalence of headache of the entire population, no gender-separate one.

In our study, we detected that more than half of the included MS patients (55.4%) suffered from headaches. This result is consistent with data from studies by Kister et al. [13] with 64%, D'Amico et al. [12] with 57.7%, Gee et al. [26] with 55.6%, Nicoletti et al. [27] with 57.4% and Putzki et al. [28] with 56.2%. In contrast Yetimalar et al. [29] with 28.5%, Boneschi et al. [30] with 35.5% and Pöllmann et al [31] with 40% quantified significantly lower prevalence rates.

Due to methodological problems it is difficult to compare accurately between theses studies, for as they refer to different sample sizes of patients, different definitions of headache (IHS-1, IHS-2, proprietary or none), a retro- or prospective design, different prevalence timeframes (four weeks, six months, one year, lifetime) and different types of recording of headache (telephone interview, postal questionnaire, personal interview at the hospital/outpatient clinic). Despite of these differences it is evident that the prevalence of headache in MS patients compared to healthy controls is significantly higher. Thus, headache could play a more important role in MS disease than previously supposed. As headache prevalence rates decrease with age [32] one could assume that the differences between MS patients with and without headaches are due to the older age of the non headache sufferers. Because of our study design we did not have the chance to compare MS patients to a healthy reference group. This is indeed a weak point but as the majority of comparable studies showed similar results in headache prevalence rates we assume that the higher prevalence rate in MS patients compared to the general population is not only based on pure age.

Additionally, MS medication such as interferons and IVIGs could be a possible reason for the higher prevalence rates. Especially RRMS patients are treated much more often with beta interferons, which are known to cause headaches in several patients. In our study MS patients with headaches received beta-interferons in 18.4%, MS patients without headaches in 17.1%. Due to the comparable rate, an increase in the overall headache prevalence seems unlikely.

The argument that MS patients treated with IVIGs are more likely to develop headaches appears legitimate. However, the prevalence rate in the literature seems with 11–81% very heterogeneous [33]. Additionally, the prevalence rate appears to be influenced by the used flow of the IVIG injection. A rate beneath 10 g/h yields lower headaches [34]. As the majority of our patients showed a significant lower flow, we tend to believe that the headache prevalence is not increased by the use of IVIGs in our sample.

Beneath the pure epidemiology the question raises to the link between MS and headaches. Several studies were performed on this subject, but the results partially provide contradictory statements.

Recent studies on the pathology of MS showed that the meninges are involved in the pathogenesis of MS with tertiary B-cell follicles manifestation in gyri. Magliozzi et al. [5], [6] examined 36 post-mortem brain tissues samples, 29 of them with a secondary chronic progressive (SCMS) and 7 with a primary progressive (PPMS) course of the MS. In 41.4% of the SCMS patients these B-cell-follicles were detected, but not in PPMS patients. MS disease was known prior to death in all patients. The course of disease was more severe in MS patients presenting B-cell-follicles. These MS patients exhibited an earlier onset and shorter periods of the disease with lethal consequences compared to the MS patients without presenting B-cell-follicles.

The B-cell-follicles discovered by Magliozzi et al. [5], [6] transfer their antibodies to manifest MS lesions leading to an inflammatory process, which is known to be interindividually different pronounced. As meningeal irritations are known to cause headaches [7], this meningeal inflammatory process could be a pathomorphological substrate of primary headaches in multiple sclerosis. Our data imply that biographical data are important factors which may correlate with the increasing prevalence of headache in MS in general. Especially age, gender, type of MS, medication of MS and EDSS play an important role. In addition, three parameters of SF-36 (physical and social functioning as well as physical pain) were significantly different between MS-groups.

MS patients with headache present significantly lower EDSS scores (table 3) and different MS courses compared to non-headache sufferers. Here, the MS patients with headaches had a significantly higher incidence of the clinically isolated syndrome (CIS) and a relapsing-remitting MS (RRMS). In contrast, in those patients who had no headache symptoms, a secondary chronic progressive type of MS was more frequent than relapsing-remitting MS. Here, it should be noted that initially 80% of MS patients suffer from relapsing-remitting MS (RRMS, [35]). Thereof approximately 50% convert into a secondary chronic progressive MS course (SCP) during the next 10–12 years after diagnosis [36]. Overall, after 20–25 years, approximately 90% of the original RRMS patients convert to an SCP [37]. Against this background, underlined by the fact that the EDSS score continuously increases with the extent of MS disease [38], headache is a symptom of the initial course of the MS. Inflammatory processes characterize this initial course.

This assumption is supported by a further analysis regarding headache medication. Assuming that only severe headaches are treated with drugs, then MS patients with headache and an appropriate headache medication are significantly younger (39.7 years vs. 44.4 years; p = 0.008, table 3) and present a lower EDSS score (2.8 vs. 3.5, p = 0.028, table 3) compared to MS patients with headache without headache medication.

What could be a possible link between the histopathological work of Magliozzi [6] and our results? Magliozzi identified ectopic B-cell follicles on the meninges of SCP patients but not in PPMS patients. In addition, the occurrence of follicles was associated with a more severe, faster and earlier course of MS. These results could be confirmed in subsequent studies [5]. In our study similar percentages of headaches amongst our SCP patients were observed. In 29 (43.3%) of the 38 SCP patients headaches occurred. Unfortunately, Magliozzi and colleagues have not yet published brain sections from CIS or RRMS patients. For considering the immunopathology of MS, the influence of the immune system decreases from the clinically isolated syndrome to the secondary chronic progressive form continuously. On the other side more degenerative signs could be observed. Therefore the degenerative nature of MS increases steadily with advancing disease course [39], [40]. On the basis of histopathological findings of Magliozzi and colleagues [6], we would expect a higher number of B-cell follicles amongst our CIS and RRMS patients, because RRMS patients suffer from headaches in 65.1% of all cases studied. Headaches may therefore play a previously underestimated role especially in SCP and RRMS patients, but could possibly be explained with a high proportion of B-cell follicles observed in postmortem brain slices. Current limitations represent the in vivo imaging of B-cell follicles in the meninges. However, with the advancement of technology in the future, these headache-inducing changes could be observed while patients are still alive.

The final analysis shows that headaches represent an important symptom of MS. This is evident both on the side of the patient, because headaches are accompanied by a significant deterioration in the quality of life, and in pathophysiology. Therefore it can be at least partially be supposed a common pathogenetic mechanism of both headaches and MS.

Our data imply that headaches are important in the initial phase of the MS, for headache patients are both significantly younger (41 years vs. 47.4 years, p = 0.001, table 2) and have a lower degree of MS disability, operationalized by the EDSS (3.0 vs. 4.2, p = 0.001, table 2) in comparison with MS patients without headache. Future studies should focus on a common pathway of headache and MS to detect possible common pathophysiological mechanisms.
